# A Novel Snaring Variant With Balloon-Assisted Stent Stabilization: The GRASP Technique

**DOI:** 10.1016/j.jaccas.2026.107275

**Published:** 2026-03-13

**Authors:** Adrien Jossart, Gregor Leibundgut, Giuseppe Colletti, Mihai Cocoi, Marouane Boukhris, Quentin Trefois, Claudiu Ungureanu

**Affiliations:** a Department of Cardiology, CHU Helora, Jolimont Hospital, La Louvière, Belgium; b Department of Cardiology, University Hospital Basel, Basel, Switzerland; c Department of Cardiology, Clinique Saint Joseph, Vivalia, Arlon, Belgium; d Department of Cardiology, “Niculae Stancioiu” Heart Institute, Cluj-Napoca, Romania; e Department of Cardiology, CHU Saint-Etienne, Saint-Etienne, France

**Keywords:** balloon catheter entrapment, guidewire entrapment, left main PCI, PCI complications, radial access, snare technique

## Abstract

**Background:**

Guidewire entrapment in previously stented peripheral vessels is a rare but potentially hazardous complication during percutaneous coronary intervention, particularly in device-related mechanisms involving interaction with implanted metallic structures.

**Case Summary:**

A 70-year-old woman undergoing left main percutaneous coronary intervention developed irreversible entrapment of a 0.014-inch coronary guidewire within a brachiocephalic trunk stent. Conventional radial retrieval failed. A bailout strategy—GRASP (guided retrieval with assisted stent protection)—combining secondary femoral access, balloon-assisted stent anchoring, and simultaneous radial snaring, enabled controlled extraction while preserving stent integrity.

**Discussion:**

Device-related entrapment carries increased risk of prosthesis deformation, vascular injury, or material rupture. Balloon-assisted stabilization with bidirectional control may facilitate safe retrieval in complex scenarios.

**Take-Home Messages:**

Distinguishing anatomical from device-related entrapment guides management. The GRASP technique may offer a safe bailout option for complex device-related retrieval.

## Visual Summary

**Figure:**
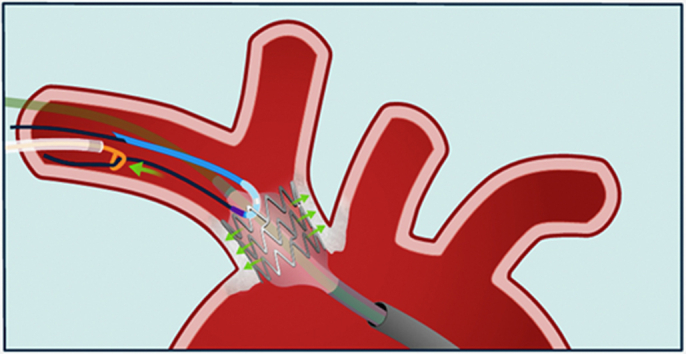

**Visual Summary:**
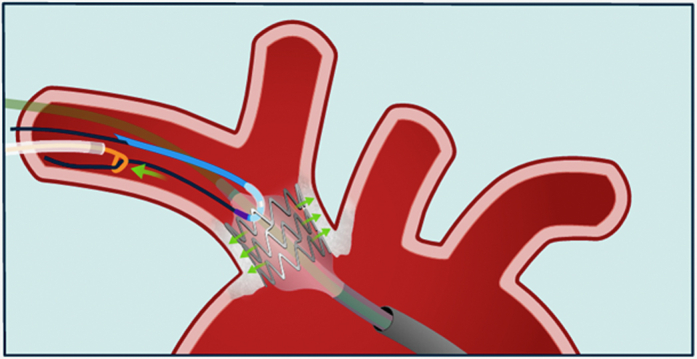
Inflation of an Armada 35 Balloon Within the Right Brachiocephalic Trunk Stent, Enabling Simultaneous Extraction of the Entrapped 0.014-inch Sion Blue Coronary Guidewire

Device entrapment remains an uncommon but serious complication during percutaneous coronary intervention (PCI). Most reported cases involve entrapment within the coronary arteries,^1^, ^2^, ^3^, ^4^ and situations in which interventional devices become lodged in noncoronary vascular structures^5^^,^^6^ are exceptionally rare and pose unique management challenges.

**Take-Home Messages**

• Device-related guidewire entrapment within previously implanted stents represents a distinct and high-risk scenario compared with anatomical entrapment, as excessive traction may lead to stent deformation, migration, vascular injury, or material rupture. Early recognition of this complication is essential to guide an appropriate retrieval strategy.
• The GRASP technique, based on balloon-assisted stent stabilization combined with bidirectional, dual-access snaring, provides controlled force distribution and may represent a safe and effective bailout option for complex device-related entrapment cases when conventional retrieval attempts fail.

Two distinct mechanisms of entrapment should be recognized: anatomical entrapment, which arises from intrinsic vessel characteristics such as tortuosity or heavy calcification; and device-related entrapment, which results from interaction with previously implanted structures such as metallic stents or prostheses. The latter carries additional procedural risks, including prosthesis deformation, migration, vascular trauma, or device rupture during extraction. Given the rarity of these events, the available literature is limited, and no standardized protocols exist to guide operators in such situations.

We present a case of guidewire entrapment within a previously implanted stent located in the brachiocephalic trunk, encountered during left main PCI via radial access. After initial retrieval attempts failed, a novel bailout strategy was employed, combining a snaring maneuver with balloon-assisted stent anchoring through secondary femoral access, allowing partial retrieval of the entrapped material.

This report describes, for the first time, a snaring variant in which peripheral balloon angioplasty was used to stabilize a brachiocephalic stent during coronary guidewire extraction.

## Case Presentation

**Figure 1:**
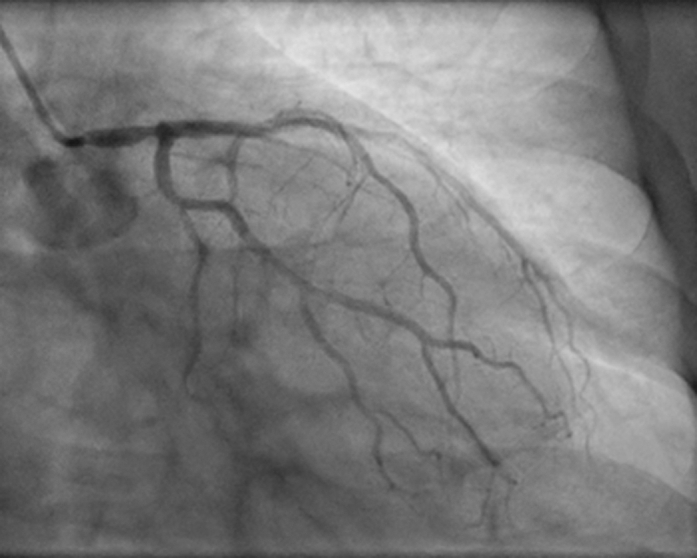
Diagnostic Coronary Angiography in the Anteroposterior Caudal View Showing Severe Ostial and Distal Left Main Stenosis

A 70-year-old woman presented with non–ST-segment elevation myocardial infarction. Diagnostic coronary angiography via the right radial artery revealed a severe left main stenosis (Figure 1) and was completed without difficulty despite a previously implanted right brachiocephalic trunk stent. After multidisciplinary heart team discussion, a deferred left main percutaneous coronary intervention was selected given the patient's advanced age and severe comorbidities, including chronic obstructive pulmonary disease.

**Figure 2:**
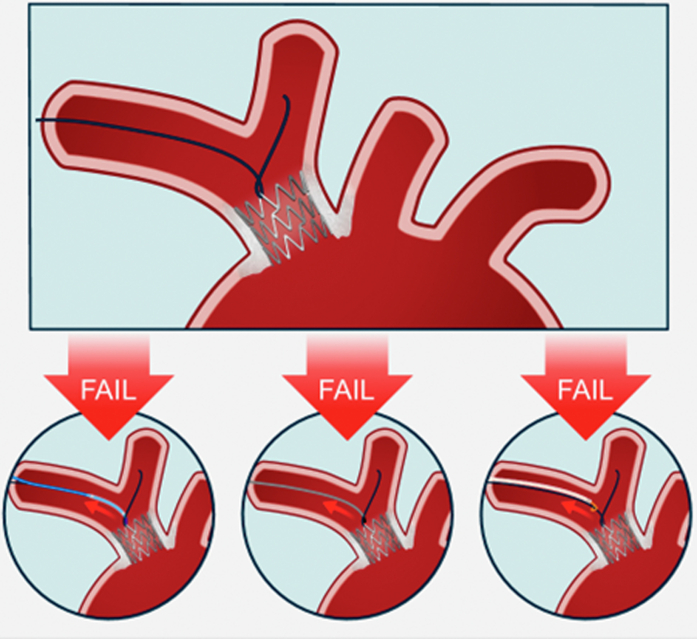
Stepwise Retrieval Attempts Performed Using a Balloon, a Mamba Flex Microcatheter, and a One Snare Device All Failed to Release the Entrapped Guidewire

**Figure 3:**
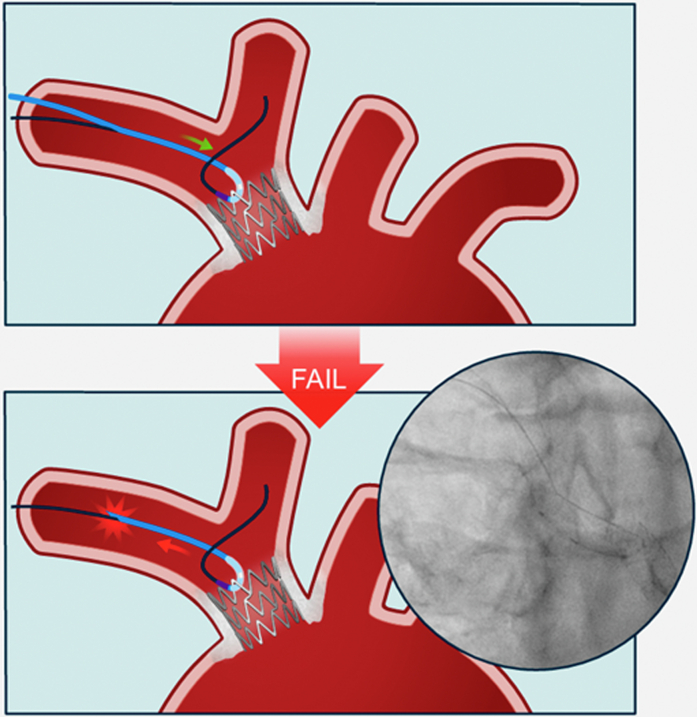
Opening of the Brachiocephalic Stent Strut With a 1.0-mm Semicompliant Ikazuchi Balloon to Facilitate Retrieval of the Entrapped 0.014-inch Sion Blue Coronary Guidewire, Complicated by Balloon Shaft Rupture in the Right Axillary Artery

During PCI, after insertion of a 7-F Glidesheath Slender (Terumo) via right radial access, we observed difficulty in crossing the right subclavian artery with a 5-F JR 4 diagnostic catheter. A 0.014-inch coronary guidewire (Sion Blue, Asahi Intecc), used to facilitate catheter passage, became entrapped within the stent, with its distal segment extending toward the right internal carotid artery. Attempts to free the guidewire by advancing a 1.0-mm low-profile semicompliant balloon (Ikazuchi, Cordis) and a microcatheter (Mamba Flex, Boston Scientific) as close as possible to the stent were unsuccessful. A 4-mm One snare (Merit Medical) was then advanced as distal as possible over the jailed guidewire. Simultaneous traction on both the guidewire and the snare resulted in visible movement of the stent and the surrounding vascular calcifications, indicating transmission of excessive pulling forces and raising concern for potential vascular injury in case of stent dislodgment (Figure 2). This approach was hence abandoned, and rather we attempted to create a larger space at the level where the coronary guidewire had probably crossed externally the first stent struts, causing its jailing. A 1.0-mm low-profile semicompliant balloon (Ikazuchi) was used for this purpose, managing to partially cross—only with its distal tip—through the stent struts. However, the balloon also became entrapped in the same spot as the guidewire, without any possibility to advance or retrieve it. During forceful traction, the balloon shaft ruptured in the right axillary artery, leaving the distal segment trapped within the vessel (Figure 3). At this stage, further direct traction on the balloon shaft or guidewire was considered high risk, with potential for stent damage or additional vascular injury.

Hence, a new strategy was adopted. First, after advancing a new 0.014-inch workhorse guidewire with a large loop tip inside the common carotid artery, the 4-mm One snare was used to capture the distal tip of the jailed guidewire and pull it back inside the subclavian artery (Video 1). Meanwhile, a second vascular access through the right femoral artery was obtained, and a 7-F, 65 cm–long Arrow sheath (Teleflex) was placed. The stent was then dilated using a peripheral 8 × 40 mm Armada balloon (Abbott) via the femoral access, but still the guidewire could not be removed. The 4-mm One snare was readvanced over the jailed guidewire. While the 8-mm balloon remained inflated within the stent to stabilize it, opposing traction forces were simultaneously applied: The guidewire and snare were pulled from one side, and countertraction was applied to the balloon from the opposite side. This “tug-of-war”–like maneuver ultimately allowed successful retrieval of the jailed guidewire (Video 2).

**Figure 4:**
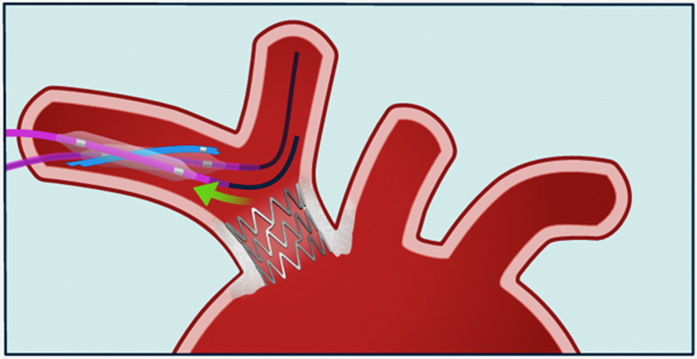
Using Two 2.0 × 20 mm Emerge Noncompliant Balloons Inflated Simultaneously Around the Retained Shaft, the Material Was Advanced Into the Right Radial Artery

However, the shaft of the ruptured small-profile balloon catheter remained in the right subclavian artery. Attempts to retrieve it using the snaring and the wire twisting techniques failed. Ultimately, two 2.0-mm Emerge noncompliant balloons (Boston Scientific) were advanced over 2 different workhorse guidewires and inflated simultaneously around the retained shaft, allowing pulldown of the ruptured balloon shaft into the right radial artery (Figure 4, Video 3). Further attempts using multiple techniques were subsequently made to achieve complete extraction of the ruptured balloon shaft. However, given important mechanical resistance that led to severe pain, the decision was made to leave the residual material in place (Video 4).

**Figure 5:**
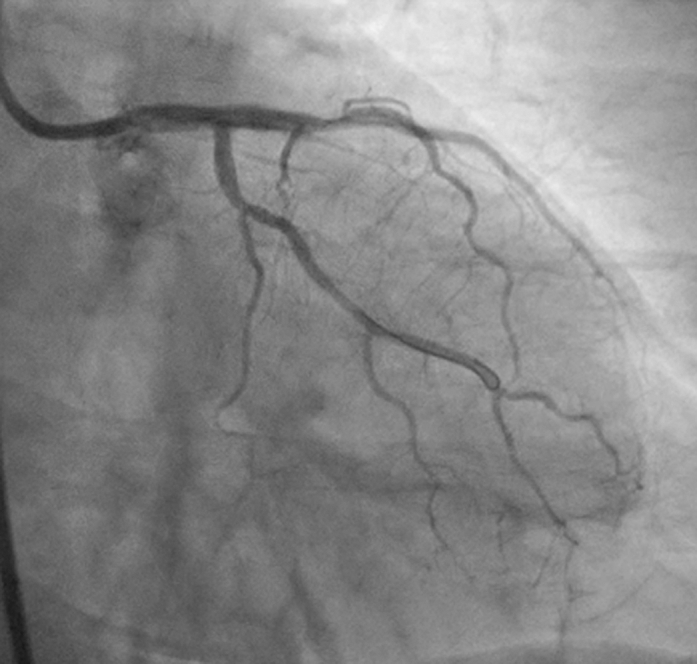
Final Angiographic Result After Left Main Percutaneous Coronary Intervention, With Implantation of a 3.0 × 33 mm Ultimaster Nagomi Stent and Proximal Optimization Using a 4.5 × 15 mm Emerge Noncompliant Balloon

Finally, left main PCI was conducted via the femoral approach, performing provisional stenting covering the left main–left anterior descending axis with a 3.0 × 33 mm Ultimaster Nagomi stent (Terumo) (Figure 5).

Surgical removal of the ruptured balloon from the radial artery was recommended to prevent thrombotic extension into the humeral artery and reduce infection risk; however, the patient declined surgery. At the 6-month follow-up, she remained asymptomatic, and arterial Doppler showed a partially thrombosed radial artery.

## Discussion

This report describes a rare case of coronary guidewire entrapment within a stent strut previously implanted in the right brachiocephalic trunk, managed using a novel bailout strategy we termed GRASP (“guided retrieval with assisted stent protection”). This technique combines distal snaring with simultaneous balloon-assisted stent stabilization to minimize the risk of stent displacement or vascular injury during extraction. In the present case, multiple predisposing factors—including diffuse vascular calcifications, vessel tortuosity, and a pre-existing stent with possibly malapposed struts—contributed to irreversible guidewire entrapment and rendered conventional radial retrieval attempts unsuccessful.

Although most reported cases of device entrapment involve the coronary arteries themselves,^1^, ^2^, ^3^, ^4^ noncoronary structures may also be implicated, posing unique retrieval challenges.^5^^,^^6^ For example, entrapment within in valvular structures has been reported, as illustrated by Sakamoto et al,^6^ who described an extraction with a laser sheath for a guidewire lodged in the tricuspid valve leaflet during right heart catheterization. This incident resulted in tricuspid regurgitation, highlighting the vulnerability of valvular structures to external mechanical forces. Closer to our case, Bentakhou et al^7^ described the entrapment of a pigtail catheter between the aortic wall and a partially deployed Acurate neo2 valve during transcatheter aortic valve implantation, where the solution involved keeping an aortic balloon inflated to stabilize the valve stent frame, allowing safe retrieval of the jailed pigtail—a concept that perfectly aligns with the strategy used in our presented case.

When excessive traction is applied during snaring—particularly at the interface between a guidewire and a stent frame—there is a substantial risk of stent migration, vascular trauma, or guidewire fracture with distal embolization.^4^ Balloon-assisted stent anchoring provides mechanical counterforce, distributes traction more evenly, and limits unidirectional pulling forces, analogous to a “tug-of-war” mechanism. This approach may reduce the risk of uncontrolled device rupture, which appears more likely in device-related than in purely anatomical entrapment scenarios.

Importantly, 2 distinct mechanisms of extra-coronary device entrapment should be recognized: anatomical entrapment, driven by vessel tortuosity or calcification, and device-related entrapment, involving interaction with previously implanted prostheses (left atrial appendage occluders,^8^ mitral repair system valves,^9^ or foramen ovale closure devices^10^). This distinction should guide retrieval strategy. In anatomical cases, the focus is to avoid vascular trauma by limiting the extraction forces and by using protective balloon catheters or microcatheters. Addressing the tortuosity of the vessels by extra-support guidewires or the vessel calcifications by angioplasty could also be indicated in specific situations. In contrast, device-related entrapment may require proactive stabilization of the implanted structure—such as with a balloon—to prevent mechanical damage during the extraction. Our case exemplifies this latter scenario, in which successful retrieval was achieved only through contralateral balloon anchoring and distal snaring using a bidirectional, “2-hands” approach. The concept of the 2-hands philosophy refers to the coordinated use of 2 distinct vascular accesses, each serving a complementary function, akin to the precision and control achieved by a surgeon using both hands during an open procedure. One access route provides stabilization and support—such as anchoring the previously implanted device or vessel using a balloon catheter—while the second access serves as the operational route for retrieval maneuvers, including snaring or traction on the entrapped device. This bidirectional approach has the potential to allow fine-tuned control of forces, limit unintended movement or deformation of vascular structures, and reduce the risk of mechanical complications such as stent migration, vessel dissection, or device rupture.

## Conclusions

Entrapment of interventional equipment in previously stented supra-aortic vessels is a rare and technically demanding scenario. When conventional retrieval techniques fail, a combination of distal snaring and balloon-assisted stent stabilization via secondary access can provide a successful bailout strategy.

## Funding Support and Author Disclosures

The authors have reported that they have no relationships relevant to the contents of this paper to disclose.

## Appendix

Video 1 Distal Snaring of the Jailed Guidewire After advancement of a new 0.014-inch workhorse guidewire with a large loop in the common carotid artery, a 4-mm One snare was used to capture the distal tip of the entrapped guidewire and reposition it back into the subclavian artery.

Video 2 Inflation of an Armada 35 Balloon Within the Right Brachiocephalic Trunk Stent, Enabling Simultaneous Extraction of the Entrapped 0.014-inch Sion Blue Coronary Guidewire

Video 3 Using Two 2.0 × 20 mm Emerge Noncompliant Balloons Inflated Simultaneously Around the Retained Shaft, the Material Was Advanced Into the Right Radial Artery

Video 4 Final Angiographic Result After Unsuccessful Complete Retrieval of the Ruptured Balloon Shaft Owing to marked mechanical resistance associated with severe patient pain, further extraction attempts were abandoned, and the residual material was left in place.
